# Epigenetic regulation of CD1d-mediated antigen presentation in B cell lymphoma

**DOI:** 10.1186/2051-1426-2-S3-P177

**Published:** 2014-11-06

**Authors:** Irina Tiper, Tonya J Webb

**Affiliations:** 1 University of Maryland School of Medicine, Baltimore, MD, USA

## 

Tumors frequently alter antigen processing and presentation by major histocompatibility complex (MHC) proteins in order to evade recognition by the immune system. CD1d, a non-polymorphic MHC class I-like molecule, presents lipid antigens to Natural killer T (NKT) cells, which have potent anti-tumor effector functions. NKT cells are able to directly lyse malignant cells and induce anti-tumor responses by modulating other immune cells. Many hematologic malignancies express CD1d and the co-stimulatory proteins needed to induce anti-tumor responses by NKT cells, yet most tumors are poorly immunogenic. Here we sought to test the hypothesis that B cell lymphomas use epigenetic mechanisms to dysregulate CD1d-mediated antigen processing and presentation leading to a functional impairment in the ability of NKT cells to recognize tumors. To assess the functional outcomes of epigenetic modulation, murine and human B cell lymphomas were pretreated with HDAC inhibitors and then we assessed their ability to process and present antigen. Pretreatment with Trichostatin A (TSA) resulted in a dose-dependent increase in CD1d-mediated NKT cell activation by lymphoma cells without altering CD1d or co-stimulatory molecule cell surface expression. Similarly, pretreatment with TSA enhanced MHC class II mediated antigen presentation to CD4+ T cells. In contrast, treatment with the more selective HDACi, MC1568, resulted in an increase in CD1d-mediated NKT cell activation, but did not enhance antigen presentation by HLA-DR4. These data indicate that HDACi differentially modulate CD1d and MHC class II-mediated antigen presentation and suggests a role for multiple HDACs in regulating antigen processing and presentation. Overall, our studies demonstrate the efficacy of HDACi in restoring NKT cell mediated anti-tumor responses and may provide the basis for an NKT cell-based immunotherapeutic strategy that not only enhances the immune response, but also increases the immunogenicity of the tumor itself.

